# Spindle cell embryonal rhabdomyosarcoma of the prostate in an adult patient – case report and review of clinicopathological features

**DOI:** 10.1186/s13000-016-0507-1

**Published:** 2016-06-29

**Authors:** Hans-Ulrich Schildhaus, Suvi Lokka, Werner Fenner, Jens Küster, Ingrid Kühnle, Ernst Heinmöller

**Affiliations:** Department of Pathology, University Medical Center Göttingen, Göttingen, Germany; Institute of Pathology Nordhessen, Germaniastr. 7, 34119 Kassel, Germany; Deparment of Urology, Nephrologisches Zentrum, Vogelsang 105, 34346 Hann. Münden, Germany; Department of Pediatric Hematology and Oncology, University Medical Center Göttingen, Göttingen, Germany

**Keywords:** Embryonal rhabdomyosarcoma, Prostate, Adults

## Abstract

**Background:**

Embryonal rhabdomyosarcoma of the prostate in an adult is a very rare event with only a few cases published. Diagnosis usually occurs with advanced disease frequently already with metastatic spread. In adults prognosis is very poor, therefore early diagnosis is crucial. To date, only three cases of spindle cell subtype of embryonal rhabdomyosarcoma of the prostate in an adult have been published.

**Case presentation:**

We report an additional case of prostatic spindle cell embryonal rhabdomyosarcoma subtype in an adult.

**Conclusions:**

We discuss relevant clinicopathological features of spindle cell embryonal rhabdomyosarcoma of the prostate in adult patients in the context of the literature.

## Background

Rhabdomyosarcomas can be classified as embryonal, alveolar and pleomorphic subtypes [1–3]. Embryonal rhabdomyosarcoma (ERMS) is the most common soft tissue sarcoma of the lower urogenital tract in children [4, 5] from birth to 15 years of age [6]. In adults ERMS is very rare. There are only individual case reports of ERMS in adulthood. In contrast to infants and children prognosis is poor in this age group [7–13]. Spindle cell rhabdomyosarcoma is considered a rare variant of ERMS that shows spindle cell morphology [9, 14]. In the latest WHO classification scheme spindle cell rhabdomyosarcoma is grouped together with sclerosing rhabdomyosarcoma [15]. ERMS shows a striking male predominance and arises most commonly from the paratesticular soft tissue, followed by the head and neck, the extremities and the genitourinary tract [16, 17]. ERMS account for only 0,3–1 % of all malignant prostate tumors [18, 19]. In pediatric patients spindle cell type of ERMS has a good prognosis and thus correct subtyping is of crucial importance for therapy and prognosis. However, the same tumor entity in adults is a very rare and aggressive tumor. In younger men it is crucial to consider this unusual differential diagnosis in order not to delay the appropriate therapy. We report a case of spindle cell embryonal rhabdomyosarcoma in the prostate of an adult patient and discuss clinicopathological features.

## Case presentation

### Clinical findings

**Fig. 1:**
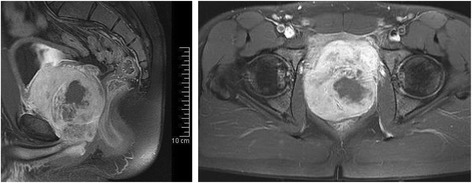
Axial (*left*) and transversal (*right*) section of magnetic resonance imaging (MRI) showing an inhomogeneous tumor mass in the prostate infiltrating the bladder wall and the seminal glands

A 25-year-old adult presented with voiding dysfunction and urinary retention. Digital rectal examination showed a rather smooth but enlarged prostate without palpable tumor mass. Ultrasound scan showed homogeneous prostate tissue and an enlarged volume of approximately 50–60 cm^3^. The clinical diagnosis was prostate adenoma. Prostate-specific antigen (PSA) level was 0,91 ng/ml and thus within normal range. After 4 weeks of therapy with the alpha_1_-receptorantagonist Tamsulosin the patient reported normal urinary function. Repeat ultrasound showed a postvoid residual volume of 150–200 ml and compared to the initial measurement further enlargement of the prostate (150 cm^3^). Magnetic resonance imaging (MRI) showed a prostatic tumor 9 × 8.5 × 7.5 cm in size with inhomogeneous tissue structure and polypoid infiltration of the bladder (Fig. 1). Six weeks after initial presentation biopsies from the prostate were taken for histological diagnosis. For staging a computed tomography (CT) scan of the lung showed bilateral pulmonary metastases. Further investigations including a FDG-PET-CT and examination of the bone marrow were negative for metastases.

### Pathological findings

**Fig. 2:**
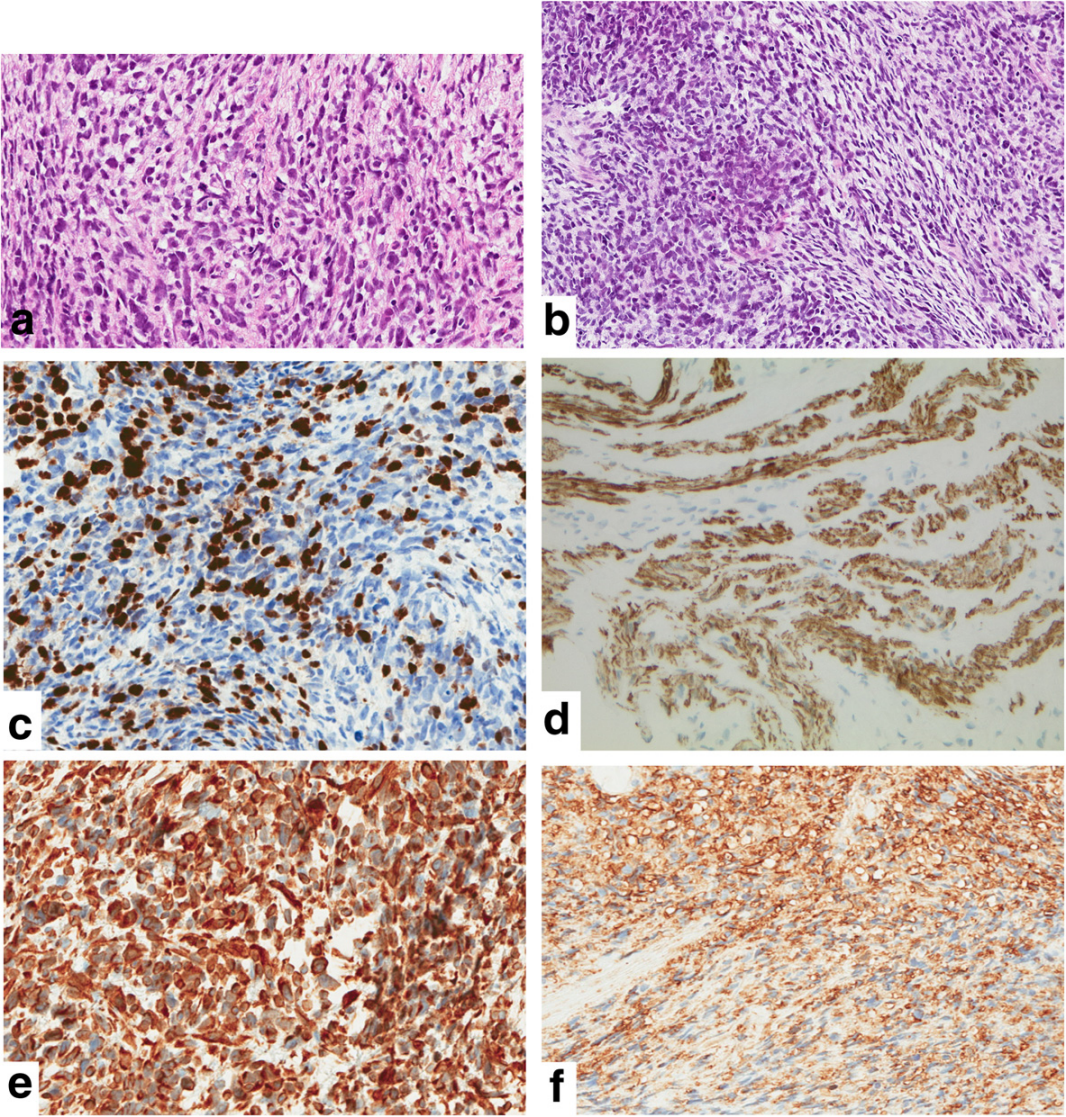
Spindle cell type of embryonal rhabdomyosarcoma showing spindle shaped tumor cells with few scattered pleomorphic cells resembling rhabdomyoblasts (**a** H&E staining, magnification x200 and **b** H&E staining, magnification x100). Tumor cells with strong nuclear staining for myogenin (**c** magnification x100) and cytoplasmatic reactivity for Desmin (**d** magnification x100), Vimentin (**e** magnification x100) and CD99 (**f** magnification x100)

Histologically, all prostate biopsies showed atypical relatively uniform tumor cells with epithelioid to spindle cell morphology arranged in an irregular fascicular proliferation pattern. The nuclei were elongated and hyperchromatic and occasionally showed prominent nucleoli. Mitotic count was up to 30 mitoses/10 high power fields. In addition, necrotic areas were present. Very few cells were identified having enlarged nuclei and voluminous eosinophilic cytoplasma resembling rhabdomyoblasts (Fig. 2a, b). Immunohistochemically, the tumor cells stained positive for vimentin, desmin, actin, myogenin and CD 99 (Fig. 2c-f). Negative staining was found for PSA, CD 45, S-100 and pan-cytokeratin. Cytogenetic analyses for *PAX3-FOXO1A* fusion and for translocation t(11;22)(q24;q12) were negative. The diagnosis of spindle cell rhabdomyosarcoma was confirmed by reference pathology (Prof. I. Leuschner, Kiel, Germany).

### Therapy and outcome

After initial staging the patient was treated according to the therapy guidelines of the CWS Cooperative Soft Tissue Sarcoma (“Weichteilsarkom”) Group (for more details see www.cws.olgahospital-stuttgart.de). Because of the presence of lung metastases the patient was classified *Stage 4 – metastatic disease*. Therapy consisted of nine cycles chemotherapy including Ifosfamide, Etoposide, Vincristine, Actinomycin D, Carboplatin and Epirubicin followed by oral chemotherapy with Trofosfamide, Etoposid and Idarubicin for 6 months. After 9 weeks of therapy disease reevaluation showed a significant reduction of the primary tumor size and of size and number of the pulmonary metastases. Since tumor resection would be accompanied by significant morbidity, radiation therapy was chosen as definitive local therapy. To better protect the surrounding structures (bladder, intestines) proton beam therapy to the primary tumor in parallel to chemotherapy was planned. Conventional radiotherapy of the bilateral pulmonary metastases was performed at the end of the intensive treatment. Unfortunately, the patient died from metastatic disease in June 2015, 17 months after initial diagnosis.

## Discussion

Spindle cell ERMS of the prostate in adults are very rare. A recently published review of the literature resulted in only 25 cases of confirmed primary ERMS of the prostate in males 18 years of age or older until October 2010 [20]. Since then, a literature search yielded five more cases of prostatic ERMS [9, 12, 13, 21, 22], resulting in a total of 30 published and confirmed primary ERMS of the prostate in adults. Of all published cases in adults so far, only three were specifically subtyped as spindle cell type of ERMS [9, 13, 22], thus, the case we present here is the fourth one published to date.

In sharp contrast to adult rhabdomyosarcoma where prognosis is generally very poor irrespective of histological subtype, in children and adolescents therapy and clinical outcome is highly dependent on histological subtype of rhabdomyosarcoma. Especially the spindle cell subtype of ERMS heralds a good prognosis and is categorized in the low risk group according to the CWS guidelines in which three risk groups are defined (low risk, standard risk, high risk). Thus, correct subtyping of soft tissue sarcomas is of utmost importance even in adults where the development of new therapy protocols may in the future be dependent on the histological subtype.

The histologic diagnosis of the spindle cell variant of ERMS is usually based on the presence of small, round to spindle-shaped tumor cells with moderate nuclear pleomorphism. Frequent findings are scattered large rhabdomyoblasts with an eccentric nucleus and striated, eosinophilic cytoplasm [9, 10].

Immunohistochemically, ERMS typically express skeletal muscle markers: Tumor cells usually stain positive for vimentin, desmin, myogenin and myoglobin [8]. However, desmin negative cases have been reported. Thus, for confirmation of the histological subtype a panel of desmin, myogenin and myoglobin is recommended [22]. A cytoplasmic staining for CD 99 is present in 15 % of all ERMS [23], in the spindle cell subtype however the positivity rate for CD 99 was reported to be 100 % [20], which is significant especially considering peripheral primitive neuroectodermal tumor (PPNET) being an important differential diagnosis.

Besides PPNET, differential diagnosis of spindle cell ERMS includes other sarcomatous tumors such as pleomorphic and alveolar rhabdomyosarcoma, leiomyosarcoma and fibrosarcoma. In addition, malignant peripheral nerve sheath tumors, spindle cell malignant melanoma, lymphomas and phyllodes tumors with focal rhabdomyoblastic differentiation have to be considered [8, 20]. Immunohistochemistry, cytogenetic and molecular pathological findings are crucial to confirm the diagnosis [8].

ERMS lack the characteristic translocation t(1;13) and t(2;13) of alveolar rhabdomyosarcoma. In contrast to ERMS, leiomyosarcomas stain negatively for myoglobin and myogenin. Fibrosarcomas do not show scattered rhabdomyoblasts in hematoxylin and eosin (H&E) staining and express neither desmin nor myogenin. In PPNET, immunohistochemical staining with myogenin is mostly negative [8]. PPNET with divergent skeletal muscle differentiation may show immunohistologic features of skeletal muscles. Here, cytogenetic analysis for translocation t(11;22)(q24;q12) which is found in most cases of PPNET but not in ERMS is of utmost importance [23]. In contrast to ERMS, malignant peripheral nerve sheath tumors express neural markers such as S-100. Malignant melanomas, unlike ERMS, stain positively with melanocytic markers [20]. Lymphomas show immunopositivity for common leukocyte antigen CD 45.

Molecular pathological diagnostics to date have not detected any specific mutations in ERMS. In some cases, a loss of heterozygosity has been described in the chromosomal region 11p15.5 [20].

Clinical symptoms usually include micturition problems like urinary retention, dysuria and seldom hematuria. Typical clinical findings include an enlarged prostate gland and normal serum value of PSA. The disease usually progresses rapidly so that diagnosis is often made at an advanced stage with presence of distant metastases [7]. Macroscopic findings often show a nodular tumor with grayish-white indurated cut surfaces [14].

In our case, a 25-year-old adolescent presented with micturition problems and an enlarged prostate gland. PSA levels were within normal range. At this age, ERMS of the prostate is rather seldom as ERMS occur more often in the lower urogenital tract in children. The most common prostate tumor in male adults is adenocarcinoma, however, this diagnosis is very rare in an age below 40 years [7]. In the age group between 20 and 40 years enlargement of the prostate gland usually is classified as benign prostatic hyperplasia and malignant disease is initially virtually never considered. Clinical differences of both tumor entities are compared in Table 1.

**Table 1:** Clinical and radiological differences between prostatic rhabdomyosarcoma and carcinoma, modified according to Waring et al. [7]

Clinical characteristics	Rhabdomyosarcoma	Carcinoma
Incidence	very rare	common
Age group	Children, rarely young adults	>40 years
Symptoms	urinary obstruction	urinary obstruction
Digital rectal examination	Enlarged, firm, smooth prostate	Hard, fixed, nodular, irregular prostate
PSA levels	normal	elevated
Course	rapid	usually slow
Ultrasound and MRI	often extensive invasion of periprostatic tissues	often localized to the prostate gland

*PSA* prostate-specific antigen, *MRI* Magnetic resonance imaging

Treatment of ERMS depends on stage of disease and includes a combination of chemotherapy, radical surgery and radiotherapy [10]. Our patient was treated according to the recommendations of the CWS-guidance for risk adapted treatment of soft tissue sarcoma and soft tissue tumors in children, adolescents, and young adults. After chemotherapy treatment he showed a good partial response. Local therapy of the tumor is a crucial element in the overall treatment.

If resection of the primary tumor would be mutilating as in our case radiotherapy is a local therapy option. 50 Gy (conventional fractionated) is considered as sufficient for rhabdomyosarcoma with residual disease following induction chemotherapy without an option for secondary resection [24].

Prognosis of ERMS in adults is generally poor. Most patients with prostatic ERMS die under therapy. Children and adolescents usually have a much better response to multimodal therapy than adults and primary surgical treatment is not standard of therapy [10, 11]. In a retrospective study Wang et al. analysed outcome of 25 adult patients with prostate sarcoma. Age more than 50 years, metastasis at presentation, and a lack of surgery with curative intent were independently predictive of an unfavorable outcome [25]. Musser et al. reviewed 38 cases of adult prostate sarcoma treated at the Memorial Sloan Kettering Cancer Center between 1982 and 2012. They found an association between histological tumor subtype and outcome: Rhabdomyosarcoma patients had worse overall and cancer-specific survival compared to leiomyosarcoma patients [26]. Latz et al. [9] describe a case of spindle cell rhabdomyosarcoma of the prostate in a 23-year-old patient who died 14 month after diagnosis being treated within the CWS 2002 P study which includes children and adolescents with soft tissue sarcoma. They retrospectively criticize that early radical surgery was not performed in the first place but the patient received radiochemotherapy. Latz et al. [9] discuss that spindle cell rhabdomyosarcoma in adults is not synonymous with rhabdomyosarcoma in childhood leaving primary radical surgical therapy as the only option for curative therapy in the absence of metastatic spread. Recently, a clinicopathological analysis of spindle cell/scerosing rhabdomyosarcoma suggested an improvement of outcome of spindle cell rhabdomyosarcoma in various locations by localized surgical treatment combined with chemotherapy in such cases with initial localized disease [22]. In the case we describe here primary radical surgery was not an option as our patient already had pulmonary metastases at the time of diagnosis. Standard therapy of prostatic rhabdomyosarcoma in adults is still to be defined.

## Conclusions

Prostatic rhabdomyosarcoma is a very rare tumor entity in adults. Prognosis is poor in contrast to children. In adults, optimal therapy may be radical surgery for early tumor stage in the absence of distant metastases. Therefore, especially in adults younger than 40 years, it is of utmost importance to consider this rare differential diagnosis in order not to delay appropriate treatment. Correct histologic subtyping of tumor should be performed to provide a basis for improvements of therapy.

## Abbreviations

CT, computed tomography; ERMS, embryonal rhabdomyosarcoma; H&E, hematoxylin and eosin; MRI, Magnetic resonance imaging; PPNET, peripheral primitive neuroectodermal tumors; PSA, prostate-specific antigen
